# Morphological analysis and immunohistochemical expression in restorations with self-adhesive resin: A randomized split-mouth design-controlled study

**DOI:** 10.4317/jced.58413

**Published:** 2021-09-01

**Authors:** Natália-Gomes de Oliveira, Marina-Torreão da Silveira, Leorik-Pereira da Silva, Keylla-Marinho-Albuquerque Barros, Laís-Maciel Costa, Marília-de Lima Soares, Marianne-de Vasconcelos Carvalho

**Affiliations:** 1 MSc, PhD student, Dental School, Universidade de Pernambuco/FOP-UPE; Camaragibe, PE, Brazil; 2 MSc, Dental School, Universidade de Pernambuco/FOP-UPE; Camaragibe, PE, Brazil; 3 MSc, PhD, Adjunct Professor, Dental School, Universidade Federal de Campina Grande (UFCG), PB, Brazil; 4 MSc, PhD, Dental School, Universidade de Pernambuco/FOP-UPE; Camaragibe, PE, Brazil; 5 MSc, PhD, Adjunct Professor, Dental School, Universidade de Pernambuco/FOP-UPE; Camaragibe, PE, Brazil

## Abstract

**Background:**

To evaluate the morphology and immunohistochemical expression of MMP-9, TIMP-3, COL-I, TNF-alfa and COX-2 in the dentinopulp complex of restorations with self adhesive composite (Vertise Flow/Kerr) compared with conventional resin composite with self-etching adhesive (Filtek Z250/3M ESPE and Clearfil SE Bond/Kuraray-Noritake).

**Material and Methods:**

A randomized, controlled, double-blind, split-mouth, two-arm clinical trial was conducted. Twenty-seven volunteers with third molars indicated for extraction received two deep class I restorations, one with each material. The morphology and immunohistochemical expression were measured at 15 days after the restorative procesures. The data were submitted to the Fisher`s Exct test (α=0.05).

**Results:**

Through morphological analysis, a slight disorganization of the odontoblastic layer was visualized, along with a slight inflammatory infiltrate in the VERT and CSEB groups (*p*< 0.05). In immunohistochemical analysis, TNF-alpha, MMP-9, TIMP-3 and COX-2 demonstrated more cases of positive pulp labeling.

**Conclusions:**

Self-adhesive resin composite and conventional resin composite with a self-etching bonding agent promoted slight alterations in the dentin-pulp complex, indicating a repair ability and reversibility of the inflammatory process against aggressions of the adhesive restorative procedure.

** Key words:**Immunohistochemistry, inflammation, randomized clinical trial, self-adhering composite, dentistry.

## Introduction

Adhesive restorations may be performed with different hybridization strategies that use complete smear layer removal by an etch-and-rinse adhesive system, or smear layer modiﬁcation by a self-etch and self-adhesive system (1). Simplification of the restorative procedure has always been a clinical aim, not only to reduce the number of operative procedures but also to decrease the number of possible errors from multiple steps (2).

One of advances in dentistry is the introduction of self-adhering flowable composite resins (SACs) which are a product of combining an all-in-one bonding system and flowable composite resin. By the incorporation of glycero-phosphate dimethacrylate functional monomer (GPDM) into the chemical composition of composite resins, the steps of direct restorative procedures are simplified. Based on manufacturer’s claim, this monomer exhibits acidic properties; it etches the tooth structure, bonds to the calcium of tooth structure, and has two functional methacrylate groups, which can copolymerize with other methacrylate monomers (3). Limited information is available on the clinical performance of SACs (4).

The composition of restorative materials may trigger an inflammatory response (5). Cytokines such as cyclooxygenase-2 (COX-2) are released as well as growth factors (tumor necrosis factor alpha - TNF-alfa) and proteolytic enzymes (such as matrix metalloproteinases – MMPs). MMPs have the capacity of degrading components of the extracellular matrix (ECM), such as collagen I (COL-I), compromising the quality of the restoration and reducing the biocompatibility of these materials (6). MMPs can be regulated by the interaction of tissue inhibitors of metalloproteinases (TIMPs). The balance between MMPs and TIMPs regulates bone remodeling rate and ECM degradation (7).

Therefore, this study is aimed to evaluate the morphology and immunohistochemical expression of MMP-9, TIMP-3, COL-I, TNF-alfa and COX-2 in the dentinopulp complex of restorations with SAC (VERT group) (Vertise Flow/Kerr) compared with conventional resin composite with self-etching adhesive (CSEB group) (Filtek Z250/3M ESPE and Clearfil SE Bond/Kuraray-Noritake). The null hypothesis tested was that no difference would be found regarding the expression of MMP-9, TIMP-3, COL-I, TNF-alpha and COX-2 between restorative techniques.

## Material and Methods

-Study design

A randomized, controlled, double-blind, split-mouth, two-arm clinical trial was conducted. This study was approved by the Ethics and Research Committee of the Universidade of Pernambuco (1.879.562) and registered by the Brazilian Registry of Clinical Trials (ReBEC) (RBR-5ggyf5).

-Population and sample size

Twenty-seven volunteers were recruited at the BucoMaxillofacial Surgery and Traumatology Service of the Dental School, Universidade de Pernambuco (FOP/UPE), PE, Brazil. Participants were aged between 18 and 40 years (mean 25.92 years) and of both sexes. A total of fifty-four third molars with an indication of extraction (orthodontic reasons) were included.

-Eligibility criteria

The study inclusion criteria were 1) two third molars indicated for extraction for orthodontic reasons; 2) healthy teeth without caries, score “0” according to the International Caries and Assessment System (ICDAS); 3) complete root development; and 4) fully erupted teeth.

Teeth without pulpal vitality or with altered pulpal vitality demonstrated using cold sensitivity tests, percussion, or palpation; the presence of pulpal calcification; and the impossibility of isolation with rubber dam were excluded.

-Randomization, allocation, and blinding

**Figure 1:**
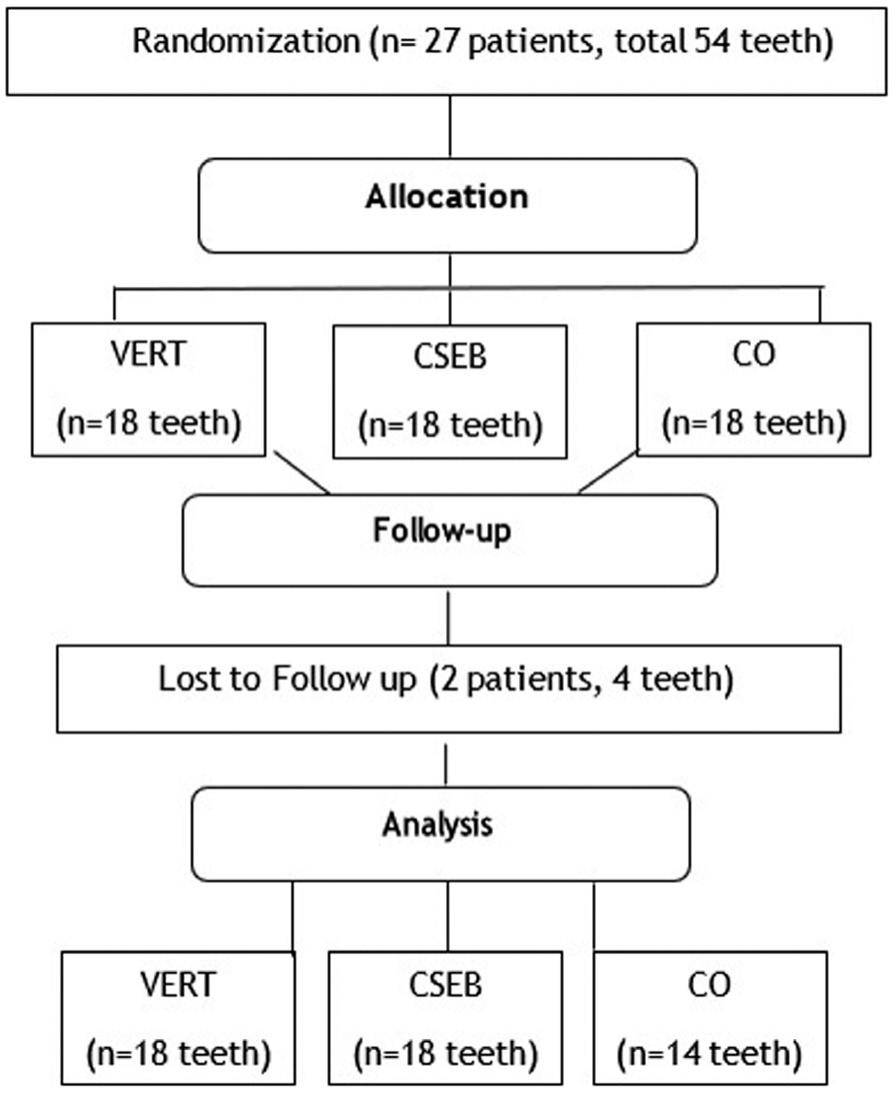
A flowchart of the study.

A total of 54 restorations in 27 volunteers were performed by the same operator (Fig. 1). Each participant received two restorations according to the experimental groups (Table 1). The restorative procedure followed the sequence of quadrants (upper right, upper left, lower left, lower right). For the choice of restorative material, a simple randomization scheme was used immediately before the bonding procedures. A researcher who was not involved in the clinical and evaluation procedures chose the restorative material to be used, by tossing a coin, where “tails” would represent the self-adhesive resin composite group (VERT), and “heads” the teeth restored using the conventional technique with prior bonding procedure (CSEB). Patients and evaluators were not aware of the type of material used for each tooth. As control group (CO), intact teeth with healthy pulp were used following the same inclusion and exclusion criteria for restored teeth. They were removed from the same patients belonging to the sample.

**Table 1:**
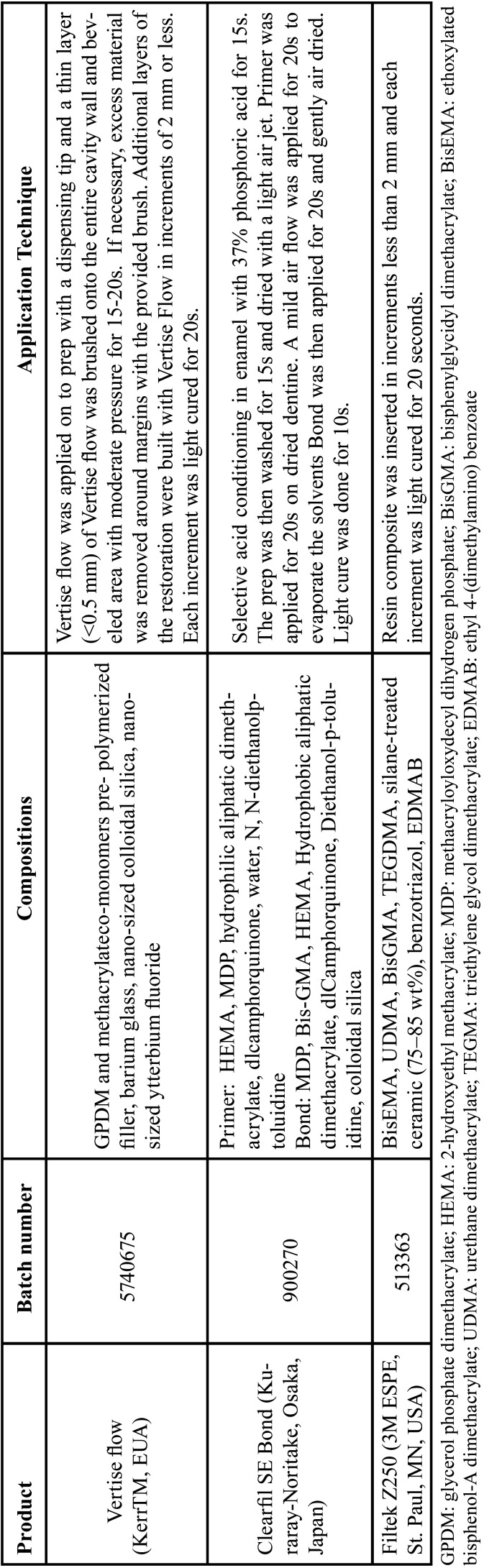
Materials used in the study according to the manufacturer’s instructions.

-Clinical procedure

•Cavity preparation

One previously calibrated operator performed all restorations. Before the restorative procedure, a periapical and a bite-wing radiograph was made. In order to standardize the cavity preparation depth (1 mm from the roof of the pulp chamber), the distance from the occlusal surface to the roof of the pulp chamber was used for measuring with the radiograph. Anesthesia was done with 2% mepivacaine with epinephrine 1:100,000 (DFL, Rio de Janeiro, RJ, Brazil), followed by tooth prophylaxis with pumice/water slurry. For cavity preparation, a long pear-shaped carbide bur (no. 245, KG Sorensen, Barueri, Brazil) was used with a highspeed handpiece with intermittent movements and abundant air/water spray. A groove was made in each bur to serve as a “stop,” limiting the depth of the preparation (1 mm from the roof of the pulp chamber). The mesiodistal extension of the cavity comprised 1/3 of the intercuspal distance. Each bur was only used once. After preparing the cavity, another bite-wing radiograph was made to confirm the cavity depth. Rubber dam isolation was done, and the cavities were disinfected with a 0.12% chlorhexidine solution. Randomization (coin-tossing) was then performed.

-Restorative procedure

The restorative materials were applied according to the manufacturers’ instructions (Table 1). All photoactivation procedures were performed with halogen light (Ultralux, DabiAtlante, Ribeirão Preto, SP, Brazil) at a power density of 800 mW/cm2, previously measured with a radiometer. After restoration placement, the occlusal contacts were evaluated with marking paper. The finishing and polishing were performed in the same session. Initially, a flame shaped fine diamond rotary instrument (no. 3118F, KG Sorensen) was used at high speed under abundant air/water spray and intermittent movement. Afterward, the restoration was polished with rubber points in decreasing order of abrasiveness using a low-speed handpiece (Microdont, São Paulo, SP, Brazil). Both restorations were done during the same clinical appointment.

-Surgical Procedure 

At the interval of 15 days, after the restorative procedure, the extractions were performed. Immediately after each extraction, 1 to 2 mm of the apical thirds of the roots were sectioned with a long diamond bur (#1065), in high speed, in intermittent movements and abundant water cooling, in order to allow the best penetration of the solution (10% formaldehyde), in which the tooth was soon submerged.

-Laboratory processing

The teeth were demineralized in 10% EDTA (Ethylenediamine tetraacetic acid). The demineralizing solution was renewed every two days, until the tissue reached the optimum demineralization to be processed, accessed with the use of microneedle tests and radiographic method. For this, the teeth were initially submitted to an orthoradial radiograph in a periapical film, representing the initial stage, that is, without decalcification. Periodically, new radiographic shots were taken in order to compare with the initial images.

-Morphological analysis

For histomorphological analysis, 5μm-thick sections were cut and stained by the hematoxylin and eosin technique. The evaluation of the tissue morphology was adapted and categorized according to the criteria of Souza, Hebling and Randall (8).

Regarding the tissue response: score 0 (none or few inflammatory cells dispersed in the pulp area); score 1 (mild inflammatory infiltrate); score 2 (Moderate infiltrate of inflammatory cells involving the coronary pulp); score 3 (Severe infiltrate of inflammatory cells involving the coronary pulp).

Regarding the organization of pulp tissue: score 0 (normal tissue morphology); score 1 (disorganized odontoblastic layer, but normal central pulp tissue); score 2 (Complete morphological disorganization of pulp tissue); score 3 (Pulp necrosis). The morphological analysis was performed comparing VERT and CSEB.

-Immunohistochemical method 

The paraffin samples were cut into 3 μm. The trilogy system (Cell Marque, Rocklin, USA) was used for deparaffinization, rehydration and antigenic recovery, in a pressure cooker and then immunohistochemical staining was performed after blocking the endogenous peroxidase by the streptavidin-biotin peroxidase method optimized by the LSAB amplification system (Strept Avidin Biotin Labeled mouse for primary antibodies - DAKO) using MMP-9, TIMP-3, TNF-alpha and COX-2 antibodies (Table 2). For COL-I, the antigen retrieval step was performed utilizing 1% pepsin solution at room temperature, for 120 minutes. As negative control, histological sections were incubated with 1% bovine serum albumin (BSA-Bovine Serum Albumin) in buffer replacing the primary antibodies. As positive control, tissue sections from breast carcinoma were used for TNF-alfa, COX-2, MMP-9 expression and placenta for TIMP-3 and COL-I expression.

**Table 2:**
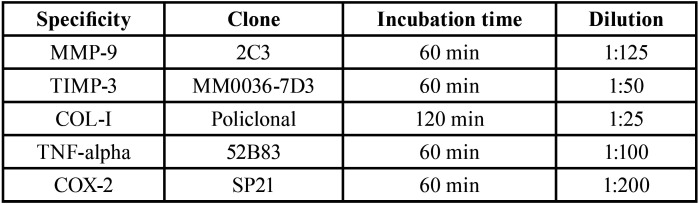
Specificity, clone, incubation time and dilution of primary antibodies.

Immunohistochemical analysis was performed by two oral pathologists. The degree of agreement between the evaluators was kappa > 0.8. Discordant cases were discussed among themselves and the evaluators came to a consensus.

This analysis was examined by light microscopy and classified considering the following aspects: presence of immunostaining (positive + or negative -); location in the tooth (dentin or pulp); location in the pulp (odontoblastic layer and central pulp region), tissue distribution (focal or diffuse) e immunostaining intensity. According to the staining intensity, the positive cases were classified in score, adapted by Alváres *et al*. (9): zero (no positive cells); mild (1-25% positive cells), moderate (25-50% positive cells) and intense (> 50% positive cells).

-Statistical analysis 

Data were analyzed descriptively by absolute and percentage frequencies. To evaluate possible differences and/or inferential associations between the expression profiles of COX-2, TNF-alpha, MMP-9, TIMP-3 and COL-I, Fisher`s Exact test used. The margin of error used in deciding statistical tests was 5%. The software used for data entry and statistical calculation was IBM & SPSS (Statistical Package for the version Social Science, version 23).

## Results

-Morphological analysis

**Figure 2:**
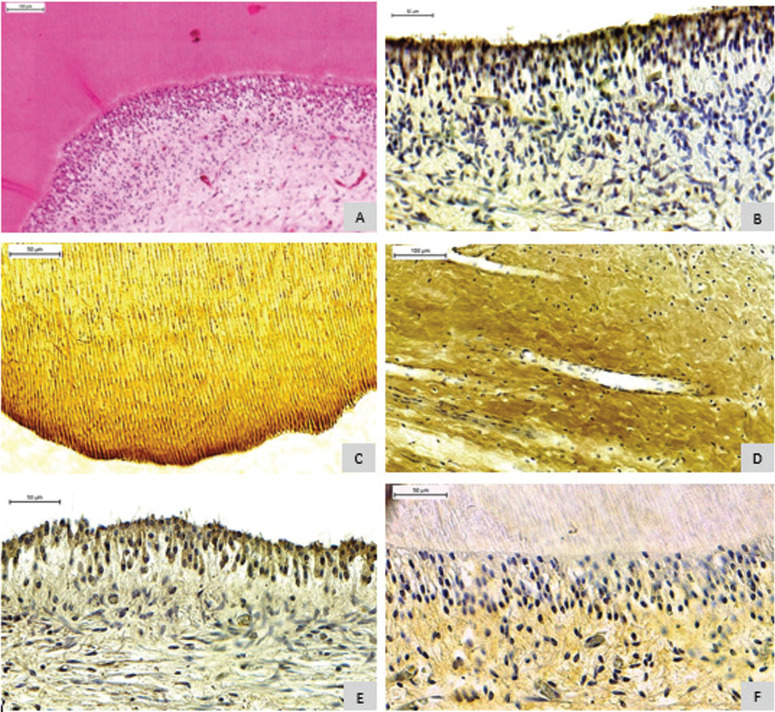
A) Morphological analysis demonstrating a disorganization of the odontoblastic layer (H.E). B) Positive immunohistochemical expression of TNF-alpha in the odontoblast layer. C) Positive immunohistochemical expression of COL-I in dentin. D) Positive immunohistochemical expression of COL-I in pulp. E) Positive immunohistochemical expression of MMP-9 in the odontoblast layer. F) Positive immunohistochemical expression of TIMP-3 in the odontoblast layer and pulp center.

Analysis of the tissue response showed a score of 0 in all groups analyzed. Regarding pulp tissue organization, score 1 was predominant VERT: 52.9% and CSEB: 64.7% (*p* < 0.05), showing the disorganized odontoblasts layer, but with normal central pulp tissue and mild inflammatory infiltrate (Fig. 2). The analysis of the tissue response as well as the organization of the pulp tissue in the CO group, showed score 0 (Table 3).

**Table 3:**
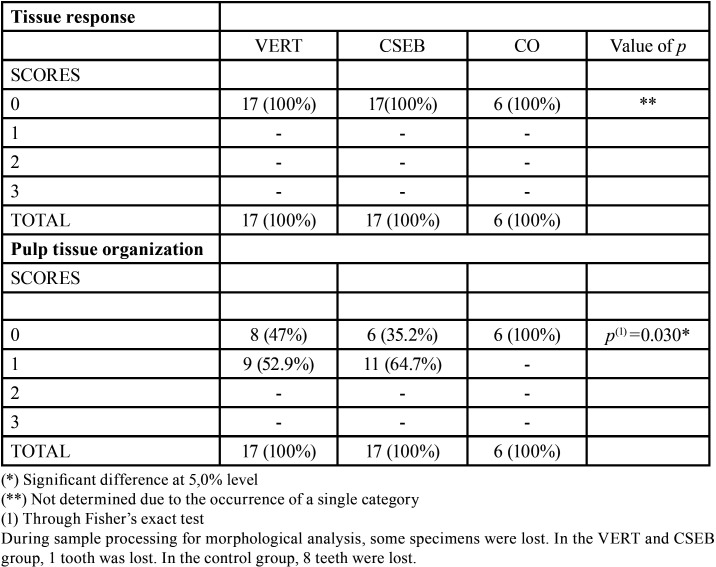
Specificity, clone, incubation time and dilution of primary antibodies.

-Immunohistochemical analysis

The immunohistochemical expression of COX-2 was negative in dentin (VERT: 93.3%; CSEB: 100%; CO: 100%) and pulp (VERT: 93.3%; CSEB: 90.9%; CO: 100%) (*p* =1.000) (Table 4).

**Table 4:**
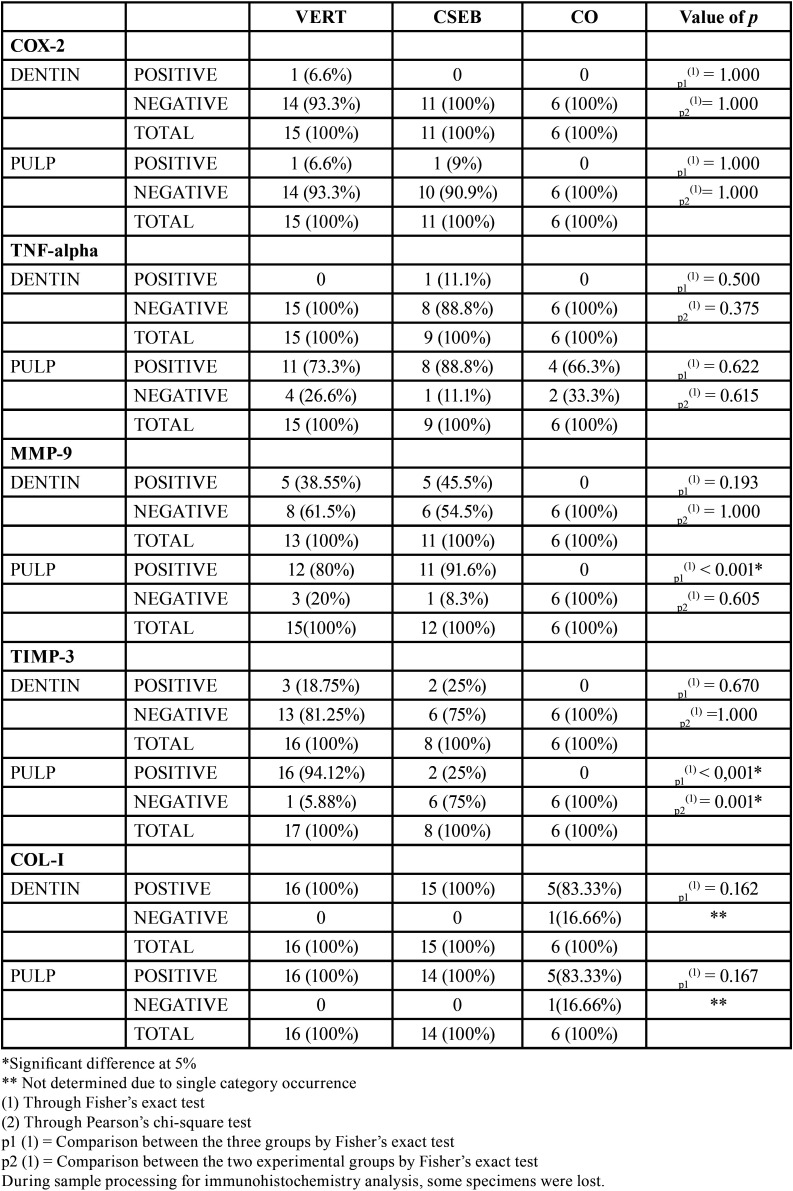
Immunohistochemical pattern (presence or absence) of TNF-alpha, COX-2, MMP-9, TIMP-3 and COL-I in pulp and dentin in VERT, CSEB and CO.

The TNF-alpha expression in the VERT and CSEB were negative in most cases for dentin (VERT: 100%; CSEB: 88.8%, CO: 100%) (*p* = 0.500). Differently, in the pulp most cases were positive (VERT: 73.3%; CSEB: 88.8%, CO:66.3%) (*p* = 0.622) (Table 4). In all cases of positivity, the marking intensity was mild. When the analysis was in pulp, the odontoblastic layer was the most frequent location. The pattern of tissue distribution of immunoreaction was diffuse (Fig. 2).

The MMP-9 expression in dentin was negative (VERT: 61.5%; CSEB: 54.5%; CO:100%) (*p* = 1.000) (Table 4) in both groups, with mild intensity and diffuse tissue distribution. In the pulp, there was positive staining in 80% VERT and 91.6% CSEB (*p* < 0.001), with higher expression and intensity in the odontoblastic layer for both groups. Staining in the CO group was negative.

Dentin staining for TIMP-3 was negative for most of the samples (VERT: 81.25%; CSEB: 75%; CO: 100%) (*p* = 1.000). In the pulp, there was positive staining only in the VERT group (94.12%). In the CSEB group, the expression was negative (75%), along with CO (100%) (*p* < 0.001), in the odontoblastic layer as well as in the center of the pulp, with mild intensity (Table 4).

COL-I expression in the dentin and in the pulp were positive for all cases in both groups (VERT and CSEB) and 83.33% in the CO group, with intense staining and diffuse tissue distribution (Table 4).

## Discussion

The null hypothesis tested was accepted as no differences were observed between the restorative materials studied. During restorative procedures, the pulp responds in different ways to aggressions resulting from both preparation and toxic elements present in the restorative materials used, which may cause some degree of inflammation (10). Chemical mediators released during the inflammatory response may trigger enzyme activation (MMPs), responsible for bone remodeling and degradation of the ECM (11). MMPs degrade exposed COL fibrils during the restorative procedure. This compromises the hybrid layer formed by the adhesive-dentin interface, reducing the bonding capacity of the restorative material (7). In the current study, the MMP-9 was more diffusely expressed in the pulp in both groups (VERT and CSEB), more specifically in the odontoblastic layer, but with mild staining intensity. In contrast, in the control group, the staining was negative. According to Mazzoni *et al*. (7,12), MMP-9 may have increased protein expression due to external stimuli by chemicals released from restorative materials. This justifies the increased expression of this protein in the groups of materials evaluated in relation to the control group. In dentin, the expression was lower than in pulp. Mazzoni *et al*. (12) studied the distribution and location of MMP-9 in demineralized and non-demineralized dentin. In both situations there was MMP-9 immunoexpression, however with greater intensity in the demineralized dentin. These findings support our results, as self-etching and self-adhesive materials promote less dentin demineralization due to the elimination of the acid etching step. According to Oliveira *et al*. (2), self-etching and self-adhesive materials disrupt the smear layer and incorporate it into the hybrid layer.

MMP-9 can degrade COL type I, IV, V, VII and X, as well as elastin and fibronectin (13). From the results found, COL-I staining was higher in both restorative groups than in the control group, both in pulp and dentin, with intense staining. The increased balance in expression between MMP-9 and TIMP-3, blocking the degradation of COL-I, could be one of the reasons. TIMP-3 was similarly expressed and comparable to MMP-9 in the different groups.

The inflammatory process is necessary for the defense of the pulp against different aggressors (such as restoration preparation and toxic elements present in restorative materials). Several biological molecules can be identified in higher concentrations in inflamed pulp tissues compared to healthy pulp tissues (14). However, in the morphological analysis all samples did not show or showed few inflammatory cells dispersed in the central pulp area. However, in most cases the odontoblastic layer was disorganized, probably due to the trauma preparation from the high-speed handpiece.

Regarding inflammatory proteins, it was possible to observe TNF-alpha immunoexpression in the pulp, both in the VERT and CSEB groups, as well as in control group. However, it is noteworthy that a greater number of positive cases were found in the restored teeth groups. Pezelj-Ribaric *et al*. (15) reported high levels of TNF-alpha in samples of teeth clinically diagnosed with irreversible pulpitis, but considerably lower expression of this cytokine in samples with normal pulps, demonstrating that this marker is related to acute inflammatory responses.

COX-2 plays an important role in pulp inflammation, being undetecTable or detected at low levels in healthy tissues and thus regulated by inflamed tissues (16,17). Huang *et al*. (18) showed expression of this mediator in human pulp cells when using two adhesive systems (Prime & Bond 2.1 and Clearfil SE Bond). Contrary to these findings, COX-2 was detected at very low levels in the present study on the dentin-pulp complex in both groups, suggesting that these materials did not provide significant levels of pulp inflammation. Kokkas *et al*. (19) also found that COX-2 activity was induced by TNF-alpha expression in human pulps, with worsening clinical symptoms of pulpitis. These data do not corroborate our findings, as it was observed that most cases were positive for TNF-alpha and negative for COX-2 in the pulp region. However, the degree of staining intensity for TNF-alpha was low. This suggests that the presence of TNF-alpha was not enough to increase COX-2 activity. The cytotoxicity of a material also depends on the thickness and permeability of the residual dentin as it determines the amount of diffusion into the pulp (20,21). In the present study, at least 1mm of remnant dentin was present when restorations were performed, and few inflammatory cells were found dispersed in the pulp region, demonstrating the reparative capacity of the dentin-pulp complex.

Thus, an extremely important feature in selecting an ideal restorative material is its compatibility and ability to interact with the dentin-pulp complex, helping to prevent pulp damage (22).

A limitation of our study was the loss of some specimens during laboratory processing for morphological and immunohistochemical analysis. Because of this, more clinical studies are needed to evaluate the morphology and immunohistochemical expression of of MMP-9, TIMP-3, COL-I, TNF-alpha and COX-2 in the dentinopulp complex of restorations with SAC.

## Conclusions

The self-etching and self-adhesive materials promoted slight alterations in the dentin-pulp complex, indicating a reversibility of the inflammatory process against aggressions of the adhesive restorative procedure.
